# To: The reality of patients requiring prolonged mechanical ventilation: a multicenter study

**DOI:** 10.5935/0103-507X.20150070

**Published:** 2015

**Authors:** Jacobo Bacariza Blanco, Antonio M. Esquinas

**Affiliations:** Intensive Care Unit, Hospital Garcia de Orta - Almada, Portugal.; Intensive Care Unit, Hospital Morales Meseguer - Murcia, Spain.

**To the Editor,**

From Averroes to Hippocrates and through Galen, Fleming or C. Venter, the history of medicine has been written as a constant stream of nonstop improvement on knowledge and clinical practice. Scientific production had not yet seen the momentum that we currently observe in the present era. With the astonishing amount of new data published in papers, research, and meta-analyses, new developments continue to arrive. However, we simultaneously face new challenges in addition to new problems demanding new solutions. Current medicine, in response to a desire for a longer life expectancy, treats more people with better care but also at a much higher cost, making many of the treatments difficult to afford and placing significant pressure on many National Health Care systems around the world. Loss et al.^(1)^ show us this reality and then analyzes what we should be doing in this regard.

Their multicenter cohort study included 93 beds from four "closed" intensive care units for a time period of 25 months (June 2008 to July 2010). The study was based on Girard et al.^(2)^ and focused on chronic critical illness, which involves paying special attention to those who require prolonged mechanical ventilation (PMV), which is defined as more than 6 hours/day during a minimum of 21 consecutive days. When compared to the patients who did not need prolonged ventilation, they concluded there was a significant increase in the death rate, admittance rates in the intensive care unit (ICU) (14.2%) and hospital (19.1%), length of stay 26.9 ± 29.3 days versus 10.3 ± 20.4 days, and in cost, which was more than 70% in the ICU and hospital.^(1)^

The solution to this matter faces two options: either to increase the national health care system budget, or to optimize the use of the tools we already have. The first option is hardly feasible, especially in light of the current economic situation and the future situation will likely not be better. The second option includes the use of noninvasive ventilation (NIV) in close association with respiratory techniques and the physiotherapy, which is probably one of the best therapeutic choices for reducing the heavy burden of the PMV patients in the ICU. Although NIV is a form of ventilation, it is always associated with fewer complications than ventilation through tracheostomy, which, in these cases, seems to be the final result for PMV patients. In avoiding the tracheostomy, we also avoid their morbidities, which in many cases are sufficient to prolong the ventilation time and require more care.

As shown in the paper, the pre-existing chronic diseases such as chronic obstructive pulmonary disease, heart failure and cancer are not only the most relevant previous conditions related to the PMV but are also three of the major indications of NIV.^(3)^ In using NIV before invasive ventilation, we reduce the need of intubation and further complications that can lead to PMV.^(4)^ In addition, we cannot forget the fourth major indication of NIV, ventilator weaning. Ventilator weaning reduces, in certain patients, the risk of failure to wean and also the need of a tracheostomy or re-intubation and further complications.

Regarding the complications that can lead to a PMV, the major ones include bacterial nosocomial sepsis (129 patients), pressure ulcers (86 patients), muscle weakness (71 patients), acute respiratory distress syndrome (37 patients), and hyperactive delirium (27 patients). These are all clinical situations that will benefit from NIV. Early extubation and NIV support will reduce the intubation time, and bacterial microfilm exposure, which precedes ventilator associated pneumonia, eventual acute respiratory distress syndrome and bacterial nosocomial sepsis. Once again, early tube withdrawal and NIV or even avoiding intubation will reduce the sedation and analgesia requirements, and subsequently, the risk of hyperactive delirium and intensive care neuropathy. In addition, NIV, when associated with daily, target planned physiotherapy^(5)^ and rehabilitating respiratory techniques, offers the possibility of early mobilization, and reduces the risk of pressure ulcers and their related comorbidities.

The remarkable study of Loss et al.^(1)^ did not include NIV data, weaning attempts using NIV, or the number of PMV patients who needed or used NIV, making it difficult to compare the NIV impact on PMV.

Finally, the major concern is the "Do Not Resuscitate" order; although it is always one of the most difficult decisions to make, it is something that we all have to face, not only once the patient is already in the ICU, but ideally many times leading up to that moment. The target is to avoid chronically critical conditions, which, in most of the cases, refers to a patient who never leaves the hospital or even passes away just a few months later at home or in any post-hospitalization support unit, especially after going through a nightmare of clinical conditions that did not translate to a good outcome.^(6)^ Furthermore, in the case of surviving the ongoing morbidity, it will unnecessarily prolong their suffering.

Unfortunately, as shown in this paper, the present severity scores fail in regard to predicting the PMV. Certainly better and more accurate decision making protocols and severity scores than the present ones would help us on this complex and unpleasant matter. Additionally, further research is required to help us reduce chronic critical illness and the unstoppable associated costs.

*Jacobo Bacariza Blanco* *Intensive Care Unit, Hospital Garcia de Orta - Almada,
Portugal.**Antonio M. Esquinas* *Intensive Care Unit, Hospital Morales Meseguer - Murcia,
Spain.*
