# Supplementary material for: To: The reality of patients requiring prolonged mechanical ventilation: a multicenter study
Source: Rev Bras Ter Intensiva. 2015 Oct-Dec;27(4):416–8. doi: 10.5935/0103-507X.20150070 (PMC4738831; doi:10.5935/0103-507X.20150070)
Supplement: Related texts

# Reply: AUTHORS' RESPONSE

**Authors:** Sergio Henrique Loss, Cassiano Teixeira

**Affiliations:** Department of Critical Care, Hospital Mãe de Deus - Porto Alegre (RS), Brazil.; Department of Critical Care, Hospital Moinhos de Vento - Porto Alegre (RS), Brazil; Department of Critical Care, Central Intensive Care Unit, Hospital Irmandade Santa Casa de Porto Alegre - Porto Alegre (RS), Brazil; Universidade Federal de Ciências da Saúde de Porto Alegre - Porto Alegre (RS), Brazil.

We appreciate the interest in our paper.^(1)^ We agree with you that prolonged mechanical ventilation as part of a chronic critical illness (CCI) could be faced as an unexpected outcome of technology. We do not allow early death in our intensive care units (ICUs), but we cannot guarantee survival with quality for this type of patient. The costs associated with this population have become large^(2)^ as well as the burden to each CCI patient and his/her family.^(3,4)^

The potential prediction of such a population early in the ICU setting is pivotal in this context. We previously published^(5)^ data from an observational cohort study in which we found that the concomitant combination of some conditions, such as admission to the ICU with sepsis, respiratory failure, abnormal mental status, and abnormal body mass index associated with suboptimal nutrition in the first week, could predict CCI with a probability of 90%. Others^(6)^ have noted that we can "see" this condition (prolonged mechanical ventilation) generating in the CCI with less time of dependence of ventilation support (2 weeks). It is hard, but we believe that it is possible to detect this type of patient earlier and offer them a more specific treatment. We agree that patients with chronic diseases (such as cancer, acquired immunodeficiency syndrome, chronic cardiac or respiratory diseases) should be submitted to noninvasive ventilation (NIV) prior to invasive mechanical ventilation, but we need more evidence to use it in acute conditions.^(7)^

We again agree with you that NIV could be a good strategy comprising this more specific treatment. Our group previously published a paper that used a NIV ventilator in tracheotomized patients with an objective of ICU-discharge.^(8)^ However, the aim of our study^(1)^ was not to study features regarding patients submitted to the NIV because in our reality, these patients either already have chronic behavior and most of them are part of a population readmitted to the ICU, or they already have CCI.

Finally, we must develop protocols to deliver the best treatment possible and reduce futility. NIV can be an excellent alternative for patients at risk of a chronic course and further studies should be conducted for this purpose.

*Sergio Henrique Loss* *Department of Critical Care, Hospital Mãe de Deus - Porto Alegre (RS),
Brazil.**Cassiano Teixeira* *Department of Critical Care, Hospital Moinhos de Vento - Porto Alegre
(RS), Brazil; Department of Critical Care, Central Intensive Care Unit,
Hospital Irmandade Santa Casa de Porto Alegre - Porto Alegre (RS), Brazil;
Universidade Federal de Ciências da Saúde de Porto Alegre - Porto Alegre
(RS), Brazil.*

## References

[1] Loss SH, Oliveira RP, Maccari JG, Savi A, Boniatti MM, Hetzel MP (2015). The reality of patients requiring prolonged mechanical
ventilation: a multicenter study. Rev Bras Ter Intensiva.

[2] Nelson JE, Cox CE, Hope AA, Carson SS (2010). Chronic critical illness. Am J Respir Crit Care Med.

[3] Nelson JE, Meier DE, Litke A, Natale DA, Siegel RE, Morrison RS (2004). The symptom burden of chronic critical
illness. Crit Care Med.

[4] Van Pelt DC, Milbrandt EB, Qin L, Weissfeld LA, Rotondi AJ, Schulz R (2007). Informal caregiver burden among survivors of prolonged
mechanical ventilation. Am J Respir Crit Care Med.

[5] Loss SH, Marchese CB, Boniatti MM, Wawrzeniak IC, Oliveira RP, Nunes LN (2013). Prediction of chronic critical illness in a general intensive
care unit. Rev Assoc Med Bras.

[6] Boniatti MM, Friedman G, Castilho RK, Vieira SR, Fialkow L (2011). Characteristics of chronically critically ill patients:
comparing two definitions. Clinics (São Paulo).

[7] Romero-Dapueto C, Budini H, Cerpa F, Caceres D, Hidalgo V, Gutiérrez T (2015). Pathophysiological basis of acute respiratory failure on
non-invasive mechanical ventilation. Open Respir Med J.

[8] Ibrahim SG, Silva JM, Borges LG, Savi A, Forgiarini LA, Teixeira C (2012). Use of a noninvasive ventilation device following
tracheotomy: an alternative to facilitate ICU discharge?. Rev Bras Ter Intensiva.
